# Measurement of gut permeability using fluorescent tracer agent technology

**DOI:** 10.1038/s41598-017-09971-y

**Published:** 2017-09-07

**Authors:** Richard B. Dorshow, Carla Hall-Moore, Nurmohammad Shaikh, Michael R. Talcott, William A. Faubion, Thomas E. Rogers, Jeng Jong Shieh, Martin P. Debreczeny, James R. Johnson, Roy B. Dyer, Ravinder J. Singh, Phillip I. Tarr

**Affiliations:** 1 MediBeacon Inc, St. Louis, MO USA; 2 Department of Pediatrics, Washington University in St. Louis School of Medicine, St. Louis, MO USA; 3 Division of Comparative Medicine, Washington University in St. Louis School of Medicine, St. Louis, MO USA; 4 Division of Gastroenterology and Hepatology, Mayo Clinic, Rochester, MN USA; 5 Immunochemical Core Laboratory, Mayo Clinic, Rochester, MN USA

## Abstract

The healthy gut restricts macromolecular and bacterial movement across tight junctions, while increased intestinal permeability accompanies many intestinal disorders. Dual sugar absorption tests, which measure intestinal permeability in humans, present challenges. Therefore, we asked if enterally administered fluorescent tracers could ascertain mucosal integrity, because transcutaneous measurement of differentially absorbed molecules could enable specimen-free evaluation of permeability. We induced small bowel injury in rats using high- (15 mg/kg), intermediate- (10 mg/kg), and low- (5 mg/kg) dose indomethacin. Then, we compared urinary ratios of enterally administered fluorescent tracers MB-402 and MB-301 to urinary ratios of sugar tracers lactulose and rhamnose. We also tested the ability of transcutaneous sensors to measure the ratios of absorbed fluorophores. Urinary fluorophore and sugar ratios reflect gut injury in an indomethacin dose dependent manner. The fluorophores generated smooth curvilinear ratio trajectories with wide dynamic ranges. The more chaotic sugar ratios had narrower dynamic ranges. Fluorophore ratios measured through the skin distinguished indomethacin-challenged from same day control rats. Enterally administered fluorophores can identify intestinal injury in a rat model. Fluorophore ratios are measureable through the skin, obviating drawbacks of dual sugar absorption tests. Pending validation, this technology should be considered for human use.

## Introduction

A cardinal function of the gut is to restrict entry of undigested food, macromolecules, and bacteria into the submucosa, lymphatics, and circulation. In the healthy gut, tight junctions between the apical surfaces of the epithelial cells blanket the inner lining of the mucosa and maintain the barrier between luminal contents and the host. In contrast, this barrier is disrupted in many different inflammatory conditions. However, it is not known if increased intestinal permeability causes, or is secondary to, gut injury. Nonetheless, it is reasonable to attempt to measure this lesion in humans to detect and quantify mucosal damage, and to use permeability as a therapeutic target. In humans, intestinal permeability is measured indirectly by determining circulating concentrations of bacterial lipopolysaccharide or antibodies to the lipopolysaccharide core moiety, and directly by dual sugar absorption tests.

Dual sugar absorption testing is a functional assessment of permeability that compares uptake into the circulation of a synthetic disaccharide, generally lactulose (MW = 342), and a monosaccharide, usually mannitol (MW = 182) or rhamnose (MW = 164). These sugars are administered together in solution by mouth. The monosaccharide traverses intact as well as disrupted epithelium, so its uptake from the lumen represents transcellular as well as paracellular transport, and reflects total gut absorptive capacity. Lactulose, which is neither actively transported across membranes nor hydrolyzed to its component monosaccharides, gains scant access to the circulation unless paracellular pathways are patent, as in intestinal inflammation. The kidneys clear the absorbed sugars as intact molecules by glomerular filtration without tubular secretion or absorption. Because the orally administered disaccharide has enhanced ability to gain entrance to the circulation if the mucosa has reduced structural integrity, elevated urinary ratios of disaccharide to monosaccharide following ingestion represent increased intestinal permeability. The ratio also controls for variable gastric emptying rates and urinary concentration conditions, as each tracer within a pair is jointly subject to the same processes in the stomach and the urinary space.

Dual sugar absorption tests are theoretically quite sound measures of intestinal permeability, but they have many technical limitations^1^. Ratios of the sugars are most commonly performed on a single specimen, but voiding is quite unpredictable, especially in children. The ideal timing for capturing the “column” of sugars filtered at the glomerulus varies between subjects and populations^2^. For children, bags must be placed over genitals to obtain urine, and adhesive failure and spillage are common. Bacteria can degrade the sugars, so to prevent post-void contamination, preservatives are required if specimens cannot be frozen immediately after acquisition, and fecal contamination of the urine must be assiduously avoided. Repeated blood or urine sampling by an indwelling venous or urinary bladder catheter might overcome these problems, but have little practical appeal. Finally, sugar assays increasingly use liquid chromatography/mass spectrometry^3^, an analytical technology that is superior to high performance liquid chromatography^4^, but which is rarely available outside research laboratories.

The limitations of dual sugar absorption tests largely relate to timing of specimen acquisition, handling of urines, and assay performance. Therefore, we asked if fluorescent tracer agents that have molecular weights similar to those of the molecules used in the dual sugar absorption test, and which are exclusively excreted by glomerular filtration, could be used to assess intestinal permeability. If fluorophores are physiologically equivalent to the sugars, they could offer advantages because their clearance can be measured through the skin, thereby obviating the need to obtain urine and to perform assays to determine relative concentrations.

Here we determined if fluorophores MB-402 and MB-301 are physiologically equivalent to the molecules used in dual sugar absorption testing of intestinal barrier function in a rat model of indomethacin-induced small bowel injury. The molecular weights of MB-402 (422) and MB-301 (198) are similar to those of the disaccharide and monosaccharides used in dual sugar absorption tests, so we postulated that these fluorophores could also measure transcellular and paracellular transport. Furthermore, these pyrazine analogs belong to a group of compounds designed for enhanced renal clearance, and therefore share excretion properties with the molecules used in the dual sugar absorption tests. Finally, the differentiating incident and emission wavelengths of MB-402 and MB-301 (see Supplementary Table S1) can be exploited to measure their systemic uptake through intact skin, eliminating the need to collect urine.

## Results

**Figure 1:**
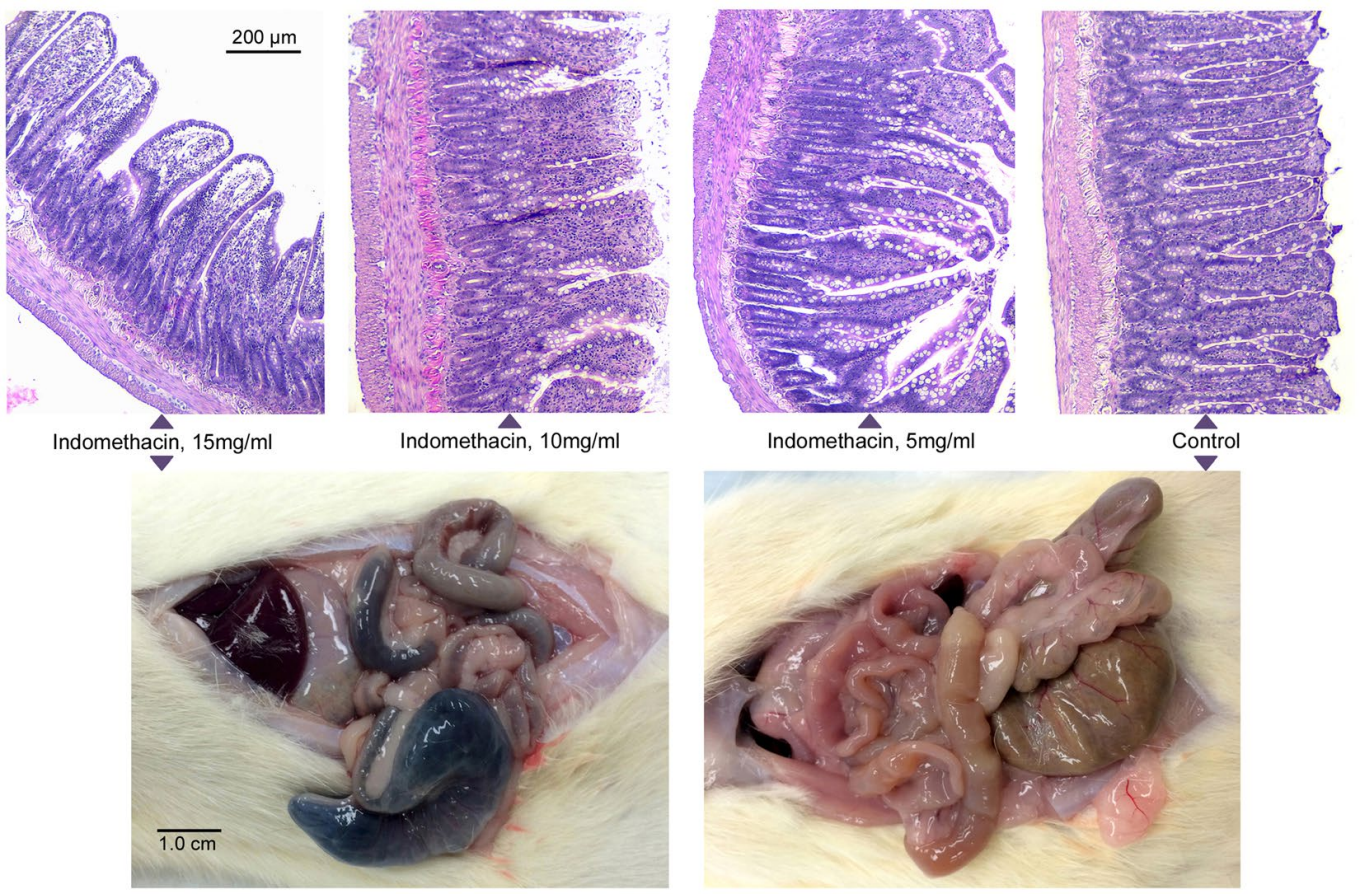
Dose-related histologic injury to distal small bowel. Top row: Hematoxylin and eosin staining of distal small bowel at necropsy after prior-day challenge with high-, intermediate-, or low- dose indomethacin or vehicle alone, as indicated. Bottom panels demonstrate transmural small bowel injury at necropsy after prior-day challenge with high-dose indomethacin (left) or normal appearance of bowel after prior-day administration of vehicle alone in a control rat (right).

High-dose (15 mg/kg) indomethacin caused severe transmural injury from the duodenum to the cecum (Fig. 1). There was diminishing visible injury to the serosa at intermediate- (10 mg/kg) and low- (5 mg/kg) doses; the serosa was normal in controls. Widened villi in the distal small bowel are seen after high- and intermediate-dose challenges, and villi are markedly shortened after high-dose challenge.

**Figure 2:**
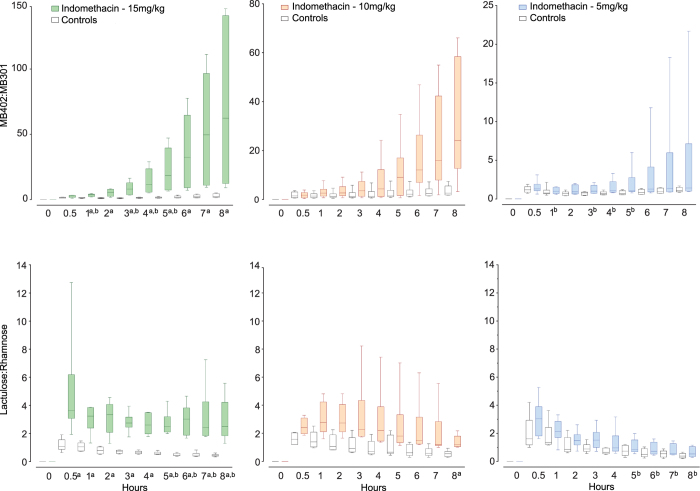
Urinary ratios of gavaged tracers. Ratios of tracers (Y-axes) in urines are plotted against time after gavage (X-axes). Note different scales on Y-axes for the fluorophores as indomethacin doses change. Box plots (generated in GraphPad Prism ver 7) portray medians and interquartile ranges with min and max whiskers for each point, for rats challenged with doses of indomethacin as indicated (colored boxes), and their same-day controls administered vehicle alone (open boxes). Fluorophore ratios are presented in the top row, with the Y-axis scales varying across challenge doses. Lactulose: rhamnose ratios are presented in the bottom row, with constant Y-axis scales. All sampling points present data from six challenged and six same day control rats, except for two time points in rats tested with the sugar tracers after intermediate dose challenge (details in statistics section). ^a^p < 0.0125 challenged rats vs. same-day control rats; ^b^p < 0.0125 high-dose vs. low-dose challenged rats. P-values for each comparison are provided in Supplementary Table S2, which also notes adjustment methodology for multiple corrections.

We detected no sugar or fluorophore tracers in baseline (T_0_) urines from 36 indomethacin-challenged and 32 control rats. The fluorophore and sugar tracers differed significantly between rats challenged with high-dose indomethacin and their same day controls at most time points (Fig. 2, Supplementary Table S2). When comparing rats given different concentrations of indomethacin on different days, the sugars and fluorophores detected statistically significant differences between high- and low-dose indomethacin approximately equally, though at different time points. The medians of the MB-402: MB301 ratios followed smooth curvilinear upward trajectories over the eight hours of sampling, and the trajectory shapes were similar and smooth across all dosing levels. In contrast, the shapes of the trajectories of urinary sugar ratios differed according to the dose of the indomethacin administered. The median sugar ratios in the urines of rats challenged with high- and intermediate-dose indomethacin fluctuated hourly.

Supplementary Fig. S1 and Supplementary Table S3 present the fold differences between tracer ratios in challenged rats and their same day controls for three different indomethacin doses, at each sampling point. The differences continuously and smoothly widen for the fluorophore ratios during these eight hours, but for the sugars, fold differences were erratic between sequential samples. At the eight-hour sampling, the median sugar ratios were 1.4-, 2.2- and 5.9-fold greater than their same-day controls after low, intermediate- and high-dose indomethacin, respectively. The corresponding spreads were more substantial when the animals were tested with MB-402 and MB-301, i.e., 1.3-, 9.3-, and 28.4–fold greater than controls.

We noted considerable inter-animal differences within challenge groups for the ratios (see Supplementary Fig. S2, Panel A), which underlie the data dispersion around the medians in Fig. 2. The MB-402: MB-301 ratios for each indomethacin dose follow curvilinear upward individual trajectories over the eight hours of sampling that resemble the aggregate trajectories for these doses, while trajectories of the individual lactulose: rhamnose ratios intersect frequently. Among the control rats, tracings of ratios of fluorophores and dual sugars are each more uniform and within a narrower range (see Supplementary Fig. S2, Panel B). However, the fluorophore ratios trend upwards, while the sugar ratios trend downwards.

**Figure 3:**
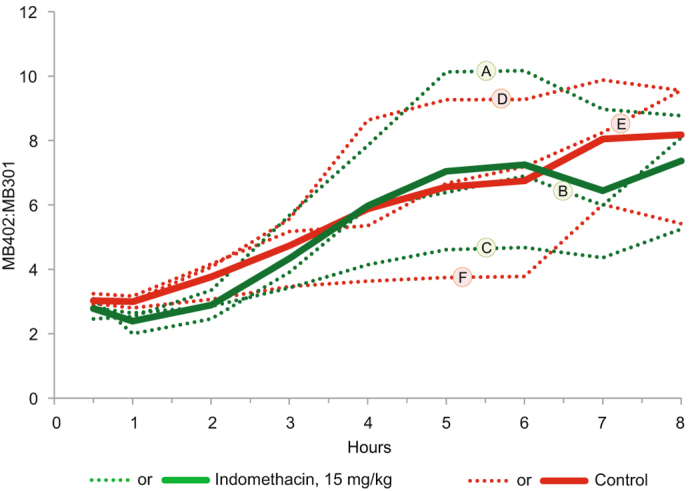
Clearance of intravenously administered fluorophores. Y-axis portrays urinary MB-402: MB-301 ratios in rats that had been gavage challenged with high-dose indomethacin or vehicle alone one day earlier (three challenged and three control rats). X-axis portrays timing of urine samples after intravenous injection of the fluorophores. Green and red dotted lines represent urinary ratios of individual challenged or individual control rats, respectively. Solid lines represent the corresponding arithmetic means of these ratios at each sampling. Letters correspond to the numbers for the same rats in Supplementary Fig. S3.

**Figure 4:**
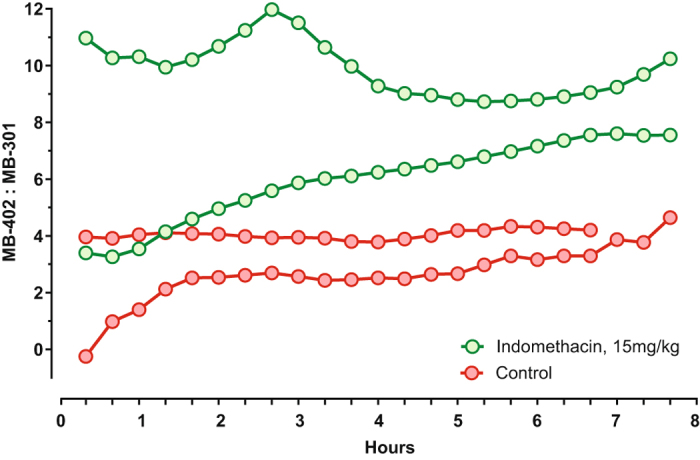
Continuous transcutaneous tracing of enterally administered MB-402 and MB-301. MB-402: MB-301 ratios (Y-axis) were determined over time (X-axis) after fluorophore gavage. One day earlier, two rats were challenged with high-dose indomethacin (green lines and circles) and two rats were challenged with vehicle alone (red lines and circles). Measurements are truncated for one control rat because it died an hour before the experiment ended.

We were concerned that the injured gut tissue might sequester the fluorophores, and/or that indomethacin diminishes renal blood flow^5^, thereby affecting clearance. However, the similar and overlapping trajectories of the ratios of the intravenously administered fluorophores in the urines in three rats challenged with high-dose indomethacin and three rats challenged with vehicle alone (Fig. 3) suggest that extra-mucosal factors cannot explain the differential ratios in Fig. 2. Within two hours of infusion, 78.1% and 75.7% of the total excreted MB-402 appeared in the urine of challenged and control rats, respectively. For MB-301, the corresponding values are 86.1% and 82.3% (see Supplementary Fig. S3). Finally, we asked if the differentiating peak fluorescence emissions of MB-402 (620 λ) and MB-301 (540 λ) can be detected transcutaneously and continuously, thereby portraying the ratios of the circulating fluorophores after gavage tracer administration. These readouts differentiated two rats challenged with high-dose indomethacin from two rats challenged with vehicle alone (Fig. 4), though the kinetics only partly resemble the kinetics generated from the 12 different rats on which the curves in each panel in Fig. 2 are based.

## Discussion

Our data preliminarily suggest that pyrazine-based fluorophores might offer advantages to dual sugar absorption testing for assessing the proportionality of indomethacin-induced injury to the proximal gastrointestinal tract. Most notably, the fluorophores appear to generate a more reliable dynamic range than that generated by the sugars across a three-fold difference in indomethacin dosing, with consistently widening spreads of values over time. These greater relative differences suggest that fluorophores might be suitable for testing interventions to improve tight junction integrity in many conditions, including childhood environmental enteropathy and dysfunction^6–11^ and associated stunting^12^, celiac disease^13^, Crohn’s Disease^14^, graft-versus-host disease^15^, non-alcoholic fatty liver disease^16^, HIV infections^17^, multi-organ failure^18^, type 1 diabetes^19^, and other^20^ disorders. Even though we do not know if increased gut permeability is a canonical lesion, or if defective barrier function merely reflects injury from other pathophysiologic processes, the associations between increased permeability and many pathologic conditions justifies pursuit of this lesion as a therapeutic target.

The data dispersion around the medians in Fig. 2 suggests that the indomethacin injury model has biologic variability, and/or there is variability in the assays. There are similar rank orders of MB-402: MB-301 ratios from hour to hour within each dosing concentration (see Supplementary Fig. S2), suggesting that the indomethacin causes more inter- than intra-rat variability, as least as determined by the fluorophores. However, sugar ratio trajectories often intersect, inferring that either assay variability, or possibly, regional effects of injury along the axis of the gut, might be responsible for the more erratic curves. Interestingly, the ratios of the sugars in the control rats intersect less extensively than the ratios of the sugars in the challenged rats, and the trajectories of the fluorophore ratios and the trajectories of the sugar ratios have different shapes, thereby raising the additional possibility that the physiologies of clearance of the fluorophores and of the sugars differ.

We found no evidence that the small bowel injury affected the ratios in the urine of intravenously injected fluorophores, but the data warrant comment. First, there is a gradually rising trajectory for both tracers over the eight hours after injection, attributed to a greater proportion of MB-402 than of MB-301 being cleared in the final six hours of sampling. Also, approximately 50% of the MB-402 and 40% of the MB-301 had not yet been excreted by the end of the experiment. Clearance is a function of the volume of distribution of the compounds and of the rate of re-entry into the plasma after distribution, and we will perform a detailed analysis of tissue distribution of the fluorophores after selecting the molecules to advance to clinical trials.

The transcutaneous tracings in the two indomethacin-challenged and the two control rats incompletely recapitulate the urinary fluorophore ratio trajectories in six challenged and six control rats (Fig. 2, upper left panel). These differences might be explained by the biologic variability of the model, the small number of animals tested, the delay between T_0_ and the start of the tracings while we affixed the sensors to the ears, or a combination of these variables. Nonetheless, these data demonstrate that ratios of circulating fluorophore can be measured through the skin.

In addition to providing instantaneous data, orally administered fluorophores might eliminate the need to collect, preserve, transport, and analyze urines, each of which is a drawback of the dual sugar absorption tests. Also, fluorophores with molecular weights greater than that of MB-402 are available and could be adapted to measure gut permeability across a gradient of injury severity. Transcutaneous detection of intravenously-administered MB-102^21^, an analog of MB-402 and MB-301, is undergoing testing in humans (NCT02098174, NCT02098187, and NCT02772276 at ClinicalTrials.gov) to measure glomerular filtration after intravenous administration of the tracer. Humans and rats excrete unchanged MB-102 in the urine within hours of intravenous injection, and this compound has not yet been associated with adverse events or toxicity. Therefore, this specimen-free technology to measure gut permeability in people could be feasible, but we await formal toxicity studies and regulatory approval before considering human use.

Many digestive disorders might be managed better by measuring gut permeability. For screening purposes, i.e., detecting increased gut permeability before clinical manifestations ensue, children at risk of environmental enteropathy and enteric dysfunction could conceivably be identified in advance of growth faltering. While some biomarkers for childhood environmental enteropathy show promise in cohort studies, they obligate recovery and analysis of urine, blood, or stool^22–25^. Small bowel biopsies, which might also be informative, are impractical in field settings. In cancer patients at risk for graft-versus-host disease of the gut, clinicians provide reactive care, increasing immune suppression only after symptoms (vomiting, diarrhea) emerge, and small bowel biopsies are diagnostic. Advanced notice of gut dysfunction could enable earlier intervention. However, these applications to human disorders require validation.

Tests of gut permeability might also play roles in the care of patients with established digestive disorders. For example, gastroenterologists increasingly endorse mucosal healing as a goal of inflammatory bowel disease care^26^, and rely on fecal tests (usually calprotectin) and colonoscopies to achieve this goal. However, improved barrier function might also serve as an indicator of mucosal healing. Evaluation of intestinal permeability has not been adapted to clinical inflammatory bowel disease practice because of the technical problems with dual sugar absorption testing, but specimen-free transcutaneous measurement of tracer ratios might overcome these limitations.

In summary, a rat model demonstrates that enterally administered fluorophores and sugars reflect gut injury in dose-dependent manners. The contours of the trajectories and the dynamic ranges of ratios of the excreted fluorophores suggest that they might be useful in clinical settings. Most particularly, if fluorophore ratios also reflect human disease severity, these tracers might make it feasible to use permeability testing to evaluate interventions designed to heal the mucosa. Based on these data, we are now adapting this specimen-free technology to measure intestinal permeability in additional models, and humans.

## Methods

### Animals

The Animal Studies Committee of the Washington University School of Medicine approved this research, and all experiments were conducted in compliance with the approval and relevant guidelines and regulations. Small bowel injury was induced using indomethacin in a rat model^27–36^. Female Sprague-Dawley rats (Charles River, Kingston, NY) are housed until challenge in the Division of Comparative Medicine facility at Washington University School of Medicine. The median weight of the animals at challenge was 245 (interquartile range 235–252) g. Rats are challenged with high- (15 mg/kg), intermediate- (10 mg/kg) or low- (5 mg/kg) dose indomethacin (Sigma, St. Louis, MO) diluted in vehicle (2% methylcellulose (Sigma, St. Louis, MO) in water), in volumes of 4 mL/kg, 18–20 hours before administering tracers. Chow but not water was withheld overnight. Two challenged and two control rats were studied on each of three different days, thereby generating groups of six challenged and six same-day control rats to compare oral tracers. For intravenous clearance studies, we studied one or two rats challenged with high-dose indomethacin and one or two control rats on each day (i.e., three rats per day on each of two days). For transcutaneous studies, two rats were challenged with high-dose indomethacin and two rats were administered vehicle only.

### Tracers

Lactulose and rhamnose (TCI America, Portland, OR) are dissolved in water. Pyrazine-class fluorescent compounds MB-402 and MB-301 (MediBeacon Inc, St. Louis, MO) are dissolved in DMSO or PBS respectively, and mixed immediately before administration by gavage. Their properties are presented in Supplementary Table S1.

### Tracer administration and sampling

Animals were anesthetized (1–4% isoflurane), and their urinary bladders were catheterized. Urine was obtained immediately before (T_0_), and 0.5, 1, 2, 3, 4, 5, 6, 7, and 8 hours after gavaging fluorophore or sugar solutions via feeding tubes or infusing fluorophores intravenously. The gavage solutions contained lactulose (50 mg/mL) and rhamnose (12.5 mg/mL) or MB-402 (10.67 mg/mL) and MB-301 (2.67 mg/mL). These solutions were administered in volumes of four mL/kg of rat body weight for the sugars (i.e., 200 mg/kg of lactulose and 50 mg/kg of rhamnose), and six mL/kg for the fluorophores (i.e., 64 mg/kg of MB-402 and 16 mg/kg of MB-301). In separate experiments, we injected one mL of a solution containing MB-402 and MB-301, corresponding to a dose of four mg/kg and one mg/kg, respectively, into the tail veins of rats that had been challenged one day before with high-dose indomethacin or vehicle alone (three rats each).

Urines are iced briefly before freezing (−80 °C), and remain frozen until assayed. Samples that are grossly bloody from catheter trauma are centrifuged. Supernatants are then iced and frozen.

### Tracer assays

Urinary lactulose and rhamnose concentrations are determined by normal phase, isocratic HPLC-tandem ESI mass spectrometry at the Mayo Clinic as a purchased service using published techniques^37, 38^. Urine samples were diluted 50-fold in deionized water before analysis. The lower limits of detection for rhamnose and for lactulose are 20 μg/mL and 15 μg/mL, respectively.

Concentrations of urinary MB-402 and MB-301 were determined using a Waters Alliance 2695 HPLC system^39^ or a Waters Acquity H Class UPLC system^40^ equipped with a column heater, sample heater/cooler, vacuum degasser, autosampler, pump capable of delivering a binary gradient, and a fluorescence detector. A RP-HPLC analytical column of Phenomenex Luna C18, 4.6 × 250 mm 5 µm, 100 Å (Phenomenex, Cat No. 00G-4252-E0, S/N H15-133556) with a Security Guard Cartridge C18 (4 × 3 mm ID, 5 μm) (Phenomenex, Cat. No. KJ0-4282) were employed to analyze urine samples via fluorescence detection. Supplementary Table S4 presents the HPLC gradient conditions used. The lower limits of detection for MB-402 and MB-301 are 50 ng/mL and 20 ng/mL, respectively.

To quantify the fluorophores, ten µL of urine was diluted 100-fold with PBS in an amber HPLC vial, mixed thoroughly on a 3-D rotator mixer, and placed in the autosampler at 5 °C. The excitation wavelength was set at 486 nm and emission wavelength was set at 600 nm. This excitation/emission combination was optimal for MB-402 quantitation and adequate for MB-301 quantitation by fluorescence. Ten µL of diluted urine sample was injected and each fluorophore was quantified using two separate sets of calibration standards prepared in 1% of rat urine/PBS. We processed and reported the data using the Waters Chromatography Data System (Empower 3 or equivalent).

All ratios represent the concentrations of the higher molecular weight molecule to the lower molecular weight molecule.

### Pathology

At experiment end, animals are euthanized and necropsied. Tissues are prepared for histopathology, and examined by light microscopy.

### Statistics

We used the unpaired non-parametric Mann-Whitney test of significance of differences between medians at each time point after T_0_ in two comparisons. First, for each group of six rats challenged with each of the three different doses of indomethacin, we compared tracer ratios in the urines to the same tracer ratios in the urines of their same day controls. Second, we compared the medians of the ratios in the urines of the rats administered high- vs. intermediate-dose, high- vs. low-dose, and intermediate- vs. low-dose indomethacin. These rats were challenged with indomethacin on separate days. Because we made four pair-wise comparisons of tracer ratios (i.e., in challenged vs. same-day controls, and the pairwise comparisons of the tracers in rats challenged with three different doses), we considered two tailed p-values < 0.0125 (i.e., 0.05 ÷ 4) as statistically significant, after adjusting for multiple comparisons.

Four rats tested with sugar tracers had dry bladders 0.5-hours after tracer gavage. Two of these four rats had been challenged with intermediate-dose indomethacin and two were same-day controls for rats challenged with the intermediate-dose of indomethacin. Hence, data from only four indomethacin-challenged and four of their corresponding control rats are presented at this sampling point. One additional rat that was challenged with intermediate-dose indomethacin died soon after the seventh hour sample was taken, so for the eighth hour data point, five challenged rats were compared to six same-day control rats, or to six rats challenged with high- or low-dose indomethacin on different days. No such problems were encountered in the 36 rats tested with the fluorophores.

### Transcutaneous determination of fluorophore ratios

After inducing anesthesia, we gavage-administered MB-402 and MB-301 to rats challenged with high-dose indomethacin and rats challenged with vehicle alone one day earlier (two rats each group). Then, animals were placed on their sides, and their ears were affixed to a glass slide with methylmethacrylate. A bifurcated light probe was placed gently against the inner surface of the ear for transcutaneous measurement of the specific fluorophore.

Baseline fluorescence (skin auto-fluorescence) was estimated by linear back-extrapolation of the first 12 minutes of measurements to the time of gavage. This value was then subtracted from all raw fluorescence measurements. The baseline-corrected fluorescence measurements were then converted into concentrations (ng/mL) using a linear fit to a series of measurements on 1 cm cuvettes. Each cuvette contained MB-402 and MB-301, at five different concentrations. All cuvettes also contained 1% Intralid to mimic the scattering properties of skin. We used a median filter to smooth concentration data from one measurement per second to three measurements per hour before computing the concentration ratios.

For articles in *Scientific Reports* reporting experiments on live vertebrates and/or higher invertebrates, the methods section must include a statement: (i) identifying the institutional and/or licensing committee approving the experiments, including any relevant details; (ii) confirming that all experiments were performed in accordance with relevant guidelines and regulations.

## Electronic supplementary material

Figure S1, S2, S3 and Tables S1, S2, S3, S4
