# Supplementary material for: Measurement of gut permeability using fluorescent tracer agent technology
Source: Sci Rep. 2017 Sep 7;7:10888. doi: 10.1038/s41598-017-09971-y (PMC5589723; doi:10.1038/s41598-017-09971-y)
Supplement: Supplementary file 1 — Figure S1, S2, S3 and Tables S1, S2, S3, S4 [file 41598_2017_9971_MOESM1_ESM.doc]

**Measurement of gut permeability using fluorescent tracer agent technology**

Richard B. Dorshow, PhD^1^, Carla Hall-Moore, BS^2^, Nurmohammad Shaikh, PhD^2^, Michael R. Talcott, DVM^3^, William A. Faubion, MD^4^, Thomas E. Rogers, PhD^1^, Jeng Jong Shieh, PhD^1^, Martin P. Debreczeny, PhD^1^, James R. Johnson, PhD^1^, Roy B. Dyer, PhD^5^, Ravinder J. Singh, PhD^5^, Phillip I. Tarr, MD^2^

^1^MediBeacon Inc., St. Louis, MO USA

^2^Department of Pediatrics, Washington University in St. Louis School of Medicine, St. Louis, MO USA

^3^Division of Comparative Medicine, Washington University in St. Louis School of Medicine, St. Louis, MO USA

^4^Division of Gastroenterology and Hepatology, Mayo Clinic, Rochester, MN USA

^5^Immunochemical Core Laboratory, Mayo Clinic, Rochester, MN USA

Correspondence to P.I.T. (tarr@wustl.edu)


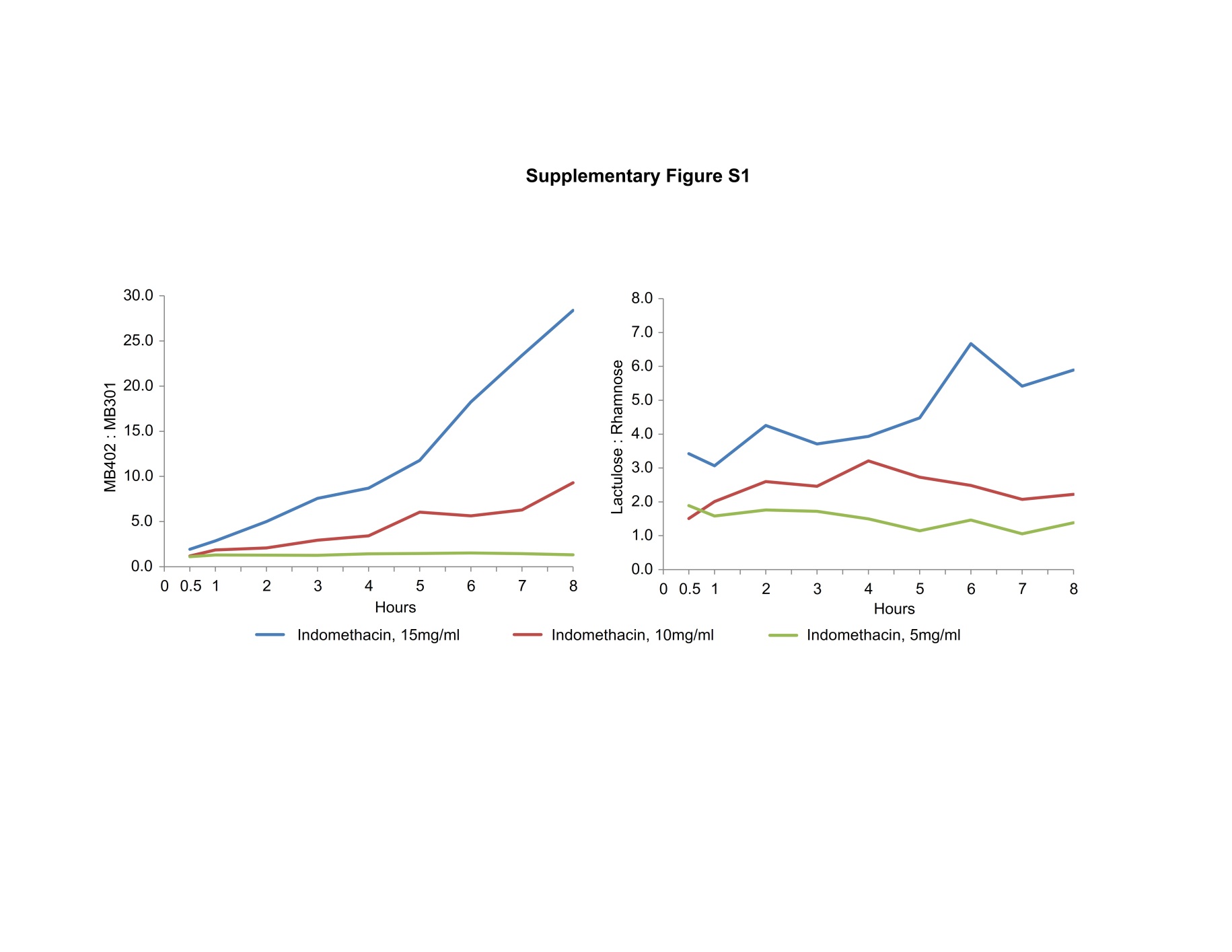


**Supplementary Figure S1. Fold-increases of tracer ratios in challenged rats over same day control tracer ratios.** Curves represent the median tracer ratios for each challenge dose divided by the median tracer ratios in the corresponding set of same day controls (Y-axes), at each time point (X-axes). Note different scales on the Y-axis in each panel. The values in Supplemental Table 3 correspond to fold differences at each point in these graphs. All points in all graphs compare six challenged to six same day control rats, except for two time points in rats tested with the sugar tracers after intermediate dose challenge (details in statistics section).


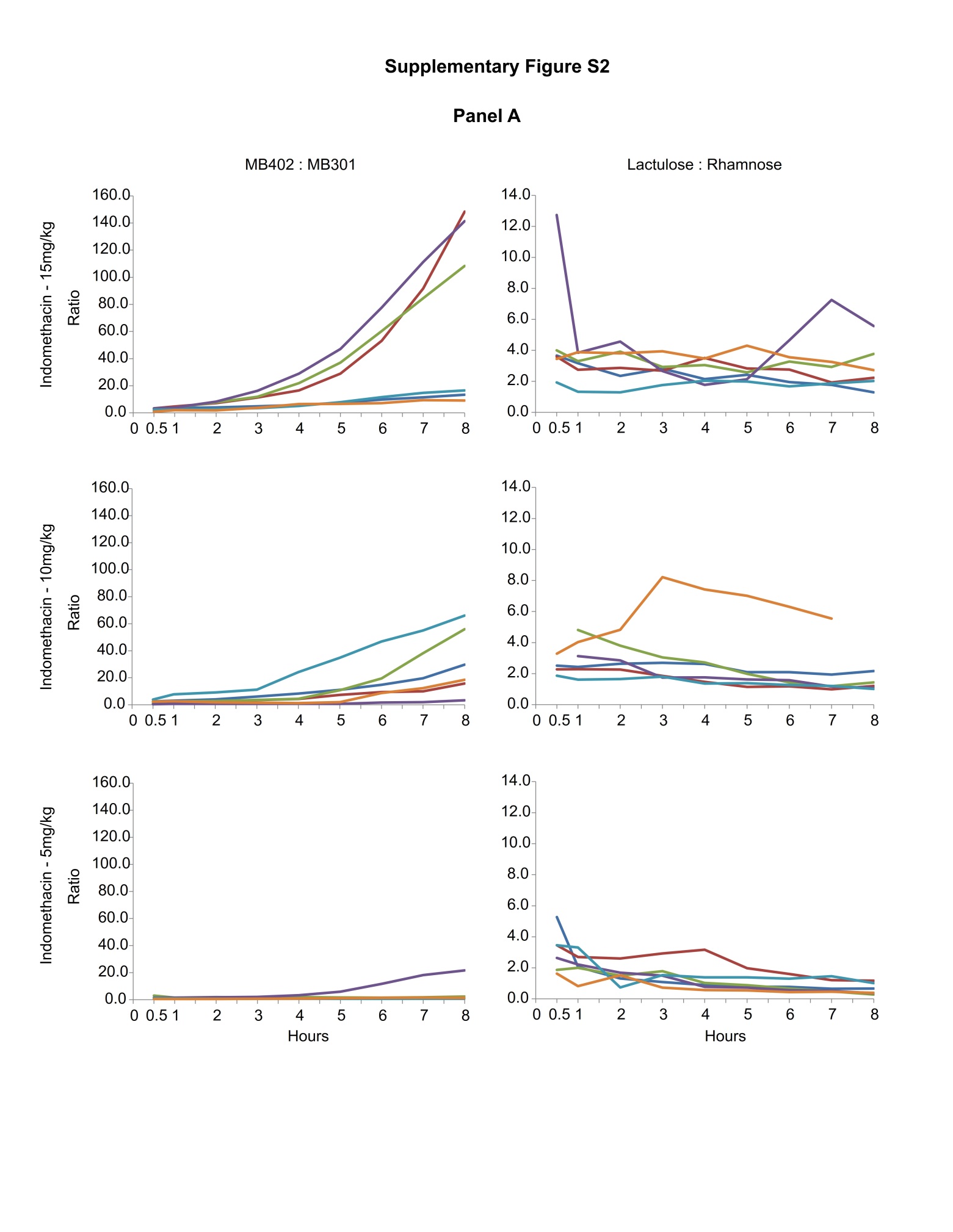


**
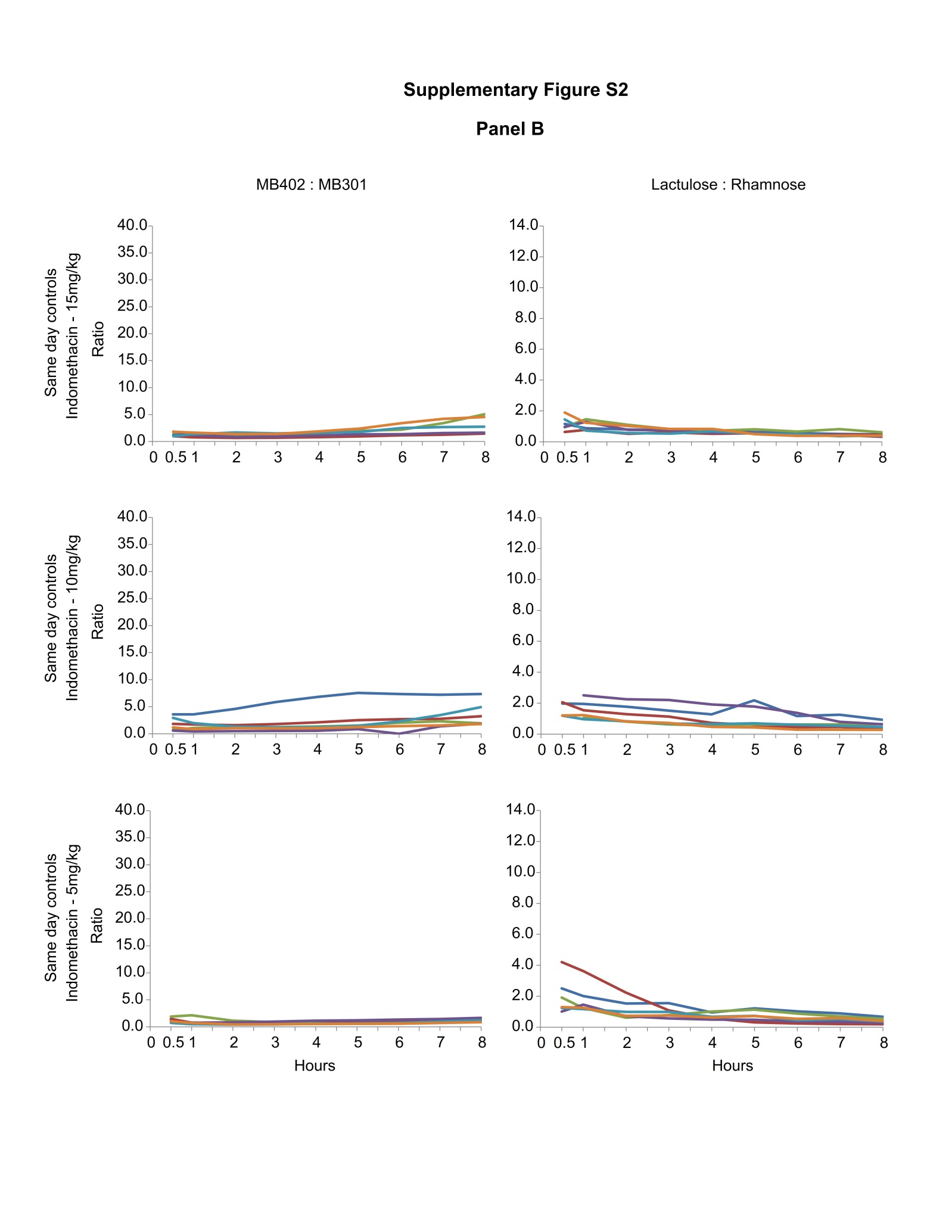
**

**Supplementary Figure S2. Individual tracer ratios.** Y-axes represent lactulose : rhamnose or MB-402 : MB-301 ratios in rats challenged with indomethacin (Panel A), or vehicle alone same day controls (Panel B), one day earlier. Note different scales in used each panel for the fluorophore tracers and the sugar tracers, and note the different scales used between panels for the fluorophore tracers. Each line follows the urinary ratios of a tracer pair in a single rat over eight hours of sampling (X-axes). The rows in panel A portray data from diminishing concentrations of indomethacin. The rows in panel B portray data from same day controls corresponding to the doses of indomethacin corresponding to the same challenged rats portrayed in Panel A. Permeability was tested with fluorophores (left column) or sugars (right column). All points in all graphs represent six challenged or six same day control rats, except for two time points in rats tested with the sugar tracers after intermediate dose challenge (details in statistics section).


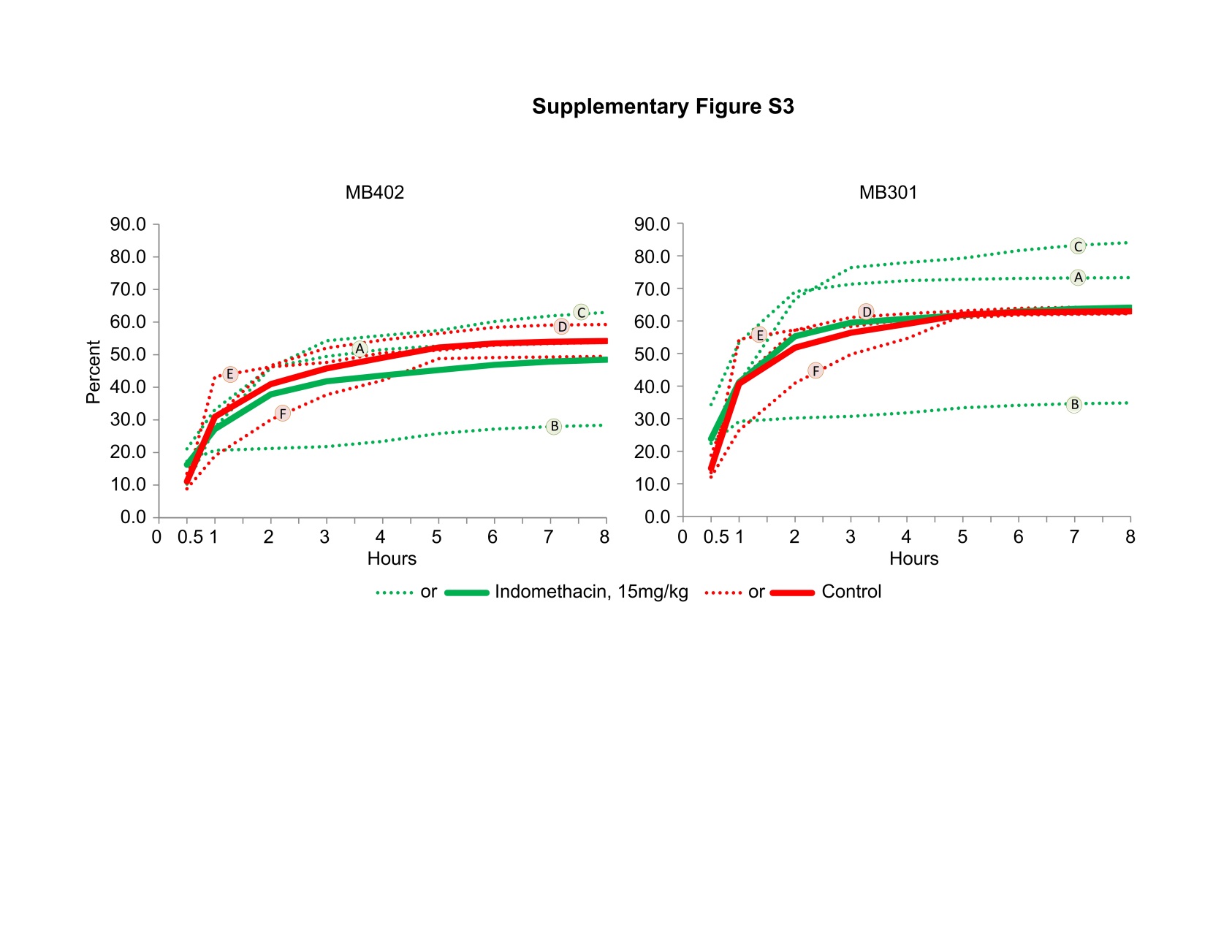


**Supplementary Figure S3. Cumulative tracer clearances after IV fluorophore administration.** Cumulative urinary recoveries as percentages of the designated fluorophore injected intravenously (Y-axes) at each sampling point (X-axes). Three rats received high-dose indomethacin and three rats received vehicle alone one day earlier. Green and red dotted lines represent data from individual challenge or control rats, respectively. Solid lines represent the corresponding arithmetic means of these ratios at each sampling. Letters correspond to the letters designating the same rats in Figure 3.

**Supplementary Table S1:** Physicochemical Properties of fluorophores

|  | **MB-402** | **MB-301** |
| --- | --- | --- |
| Structure |  |  |
| Molecular weight | 422 | 198 |
| Light absorption maximum | 500 λ | 405 λ |
| Light emission maximum | 620 λ | 540 λ |

**Supplementary Table S2:** Significance of pair-wise comparisons of medians.

| **Tracers** | **Comparison** | **0.5 hr** | **1 hr** | **2 hr** | **3 hr** | **4 hr** | **5 hr** | **6 hr** | **7 hr** | **8 hr** |
| --- | --- | --- | --- | --- | --- | --- | --- | --- | --- | --- |
| MB-402  and  MB-301 | High-dose vs. same day controls | 0.065 | 0.005^a^ | 0.005^a^ | 0.005^a^ | 0.005^a^ | 0.005^a^ | 0.005^a^ | 0.005^a^ | 0.005^a^ |
|  | Intermediate-dose vs. same day controls | 0.936 | 0.298 | 0.173 | 0.173 | 0.230 | 0.173 | 0.031 | 0.031 | 0.013 |
|  | Low-dose vs. same day controls | 0.873 | 0.471 | 0.230 | 0.128 | 0.066 | 0.093 | 0.093 | 0.230 | 0.199 |
| Lactulose  and Rhamnose | High-dose vs. same day controls | 0.005^a^ | 0.008^a^ | 0.005^a^ | 0.005^a^ | 0.005^a^ | 0.005^a^ | 0.005^a^ | 0.005^a^ | 0.005^a^ |
|  | Intermediate-dose vs. same day controls | 0.230 | 0.031 | 0.013 | 0.020 | 0.020 | 0.020 | 0.013 | 0.031 | 0.008^a^ |
|  | Low-dose vs. same day controls | 0.173 | 0.378 | 0.261 | 0.128 | 0.128 | 0.336 | 0.230 | 0.521 | 0.336 |
| MB-402  and  MB-301 | High- vs. intermediate-dose indomethacin | 0.378 | 0.378 | 0.471 | 0.128 | 0.128 | 0.298 | 0.298 | 0.378 | 0.575 |
|  | Intermediate- vs. low-dose indomethacin | 0.936 | 0.054 | 0.045 | 0.128 | 0.128 | 0.093 | 0.020 | 0.020 | 0.020 |
|  | High- vs. low-dose indomethacin | 0.092 | 0.005^a^ | 0.013 | 0.005^a^ | 0.005^a^ | 0.005^a^ | 0.020 | 0.020 | 0.020 |
| Lactulose  and Rhamnose | High- vs. intermediate-dose indomethacin | 0.689 | 0.986 | 0.689 | 0.810 | 0.378 | 0.109 | 0.128 | 0.109 | 0.030 |
|  | Intermediate- vs. low-dose indomethacin | 0.810 | 0.230 | 0.020 | 0.045 | 0.066 | 0.045 | 0.066 | 0.109 | 0.030 |
|  | High- vs. low-dose indomethacin | 0.170 | 0.128 | 0.066 | 0.066 | 0.031 | 0.006^a^ | 0.005^a^ | 0.005^a^ | 0.005^a^ |
| Median ratios of each tracer pair are compared at each sampling point, as indicated. Pair-wise comparisons are between challenged rats and same day control rats (top six rows), and between high- and intermediate-dose, intermediate- and low-dose, and high- and low-dose indomethacin-treated rats, challenged on different days.  ^a^Two-tailed P-values < 0.0125 (after correcting for multiple comparisons) considered statistically significant. | | | | | | | | | | |

**Supplementary Table S3:** Fold ratios over controls

| **Comparison** | **0.5 hr** | **1 hr** | **2 hr** | **3 hr** | **4 hr** | **5 hr** | **6 hr** | **7 hr** | **8 hr** |
| --- | --- | --- | --- | --- | --- | --- | --- | --- | --- |
| ***High-dose Indomethacin challenge*** | | | | | | | | | |
| MB-402 and MB-301 | 1.9 | 2.9 | 5.0 | 7.6 | 8.7 | 11.8 | 18.3 | 23.4 | 28.4 |
| Lactulose and Rhamnose | 3.4 | 3.1 | 4.3 | 3.7 | 3.9 | 4.5 | 6.7 | 5.4 | 5.9 |
| ***Intermediate-dose Indomethacin challenge*** | | | | | | | | | |
| MB-402 and MB-301 | 1.2 | 1.9 | 2.1 | 2.9 | 3.4 | 6.0 | 5.6 | 6.3 | 9.3 |
| Lactulose and Rhamnose | 1.5 | 2.0 | 2.6 | 2.5 | 3.2 | 2.7 | 2.5 | 2.1 | 2.2 |
| ***Low-dose Indomethacin challenge*** | | | | | | | | | |
| MB-402 and MB-301 | 1.1 | 1.3 | 1.3 | 1.3 | 1.4 | 1.5 | 1.5 | 1.5 | 1.3 |
| Lactulose and Rhamnose | 1.9 | 1.6 | 1.8 | 1.7 | 1.5 | 1.1 | 1.5 | 1.1 | 1.4 |
| Values are the medians of each tracer pair ratio in challenged rats divided by the medians of the ratios in their same-day controls. These data correspond to tracings in Supplementary Figure 1. | | | | | | | | | |

**Supplementary Table S4:** HPLC Gradient Conditions

| Time (min.) | Flow (mL/min) | MPA (%)  0.1% TFA/H_2_O | MPB (%)  0.1% TFA/ACN | Gradient Curve |
| --- | --- | --- | --- | --- |
| 0.00 | 1.0 | 90 | 10 | 6 |
| 3.00 | 1.0 | 90 | 10 | 6 |
| 10.00 | 1.0 | 50 | 50 | 6 |
| 10.05 | 1.6 | 10 | 90 | 6 |
| 12.00 | 1.6 | 10 | 90 | 6 |
| 12.05 | 1.0 | 90 | 10 | 6 |
| 15.00 | 1.0 | 90 | 10 | 6 |
